# Performance on a probabilistic inference task in healthy subjects receiving ketamine compared with patients with schizophrenia

**DOI:** 10.1177/0269881111435252

**Published:** 2012-09

**Authors:** Simon Evans, Basil Almahdi, Pervez Sultan, Imrat Sohanpal, Brigitta Brandner, Tracey Collier, Sukhi S Shergill, Roman Cregg, Bruno B Averbeck

**Affiliations:** 1 Sobell Department of Motor Neuroscience and Movement Disorders, Institute of Neurology, UCL, London, UK; 2 University College Hospital, London, UK; 3 Cognition Schizophrenia and Imaging Lab, Department of Psychiatry, Institute of Psychiatry, Kings College London, London, UK; 4 Laboratory of Neuropsychology, National Institute of Mental Health, National Institutes of Health, Bethesda, MD, USA

**Keywords:** Ketamine, decision making, schizophrenia

## Abstract

Evidence suggests that some aspects of schizophrenia can be induced in healthy volunteers through acute administration of the non-competitive NMDA-receptor antagonist, ketamine. In probabilistic inference tasks, patients with schizophrenia have been shown to ‘jump to conclusions’ (JTC) when asked to make a decision. We aimed to test whether healthy participants receiving ketamine would adopt a JTC response pattern resembling that of patients. The paradigmatic task used to investigate JTC has been the ‘urn’ task, where participants are shown a sequence of beads drawn from one of two ‘urns’, each containing coloured beads in different proportions. Participants make a decision when they think they know the urn from which beads are being drawn. We compared performance on the urn task between controls receiving acute ketamine or placebo with that of patients with schizophrenia and another group of controls matched to the patient group. Patients were shown to exhibit a JTC response pattern relative to their matched controls, whereas JTC was not evident in controls receiving ketamine relative to placebo. Ketamine does not appear to promote JTC in healthy controls, suggesting that ketamine does not affect probabilistic inferences.

## Introduction

Patients with schizophrenia have been shown to behave differently to controls on tasks involving probabilistic decision making, with patients tending towards a ‘jumping to conclusions’ (JTC) style of reasoning (Garety et al., 1991; Huq et al., 1988; Moritz and Woodward, 2005; Mortimer et al., 1996). Typically this has been demonstrated using the ‘urn’ or ‘beads’ task, first proposed by Phillips and Edwards (1966). This simple task involves two containers (‘urns’) containing a large number of different coloured beads in differing ratios. One urn might contain 85% blue beads and 15% red beads, the other 85% red beads and 15% blue beads; the participant is informed of these proportions, although the containers are hidden from view. The experimenter then presents a series of beads one at a time to the participant, apparently having chosen one of the urns to draw from (but actually presenting a pre-prepared sequence of beads). After the participant is shown each bead they are asked if they want to guess which urn the beads are being drawn from, or see another bead. Huq et al. (1988) showed that deluded patients with schizophrenia required fewer draws before claiming to know which urn had been chosen, compared with controls. This tendency to accept hypotheses prematurely has been proposed to be critical in encouraging delusion formation by contributing to erroneous inferences (Garety, 1991). JTC can be detected reliably in delusional individuals (Fine et al., 2007). However, JTC also appears to be present in patients with schizophrenia independent of whether delusions are present. Many early studies did not include a non-deluded schizophrenia group as a control, but recent studies have shown similar levels of JTC in both deluded and non-deluded patients using the beads task (Menon et al., 2006; Moritz and Woodward, 2005; Mortimer et al., 1996). Peters and Garety (2006) tested patients twice, once when actively deluded, and then again when in remission. The JTC bias was found to be stable. Similarly, successful antipsychotic treatment is not associated with a reduction in JTC (Menon et al., 2008). This indicates that JTC might be a trait marker for schizophrenia. Consistent with this, Van Dael et al. (2006) found evidence of a JTC response pattern in first-degree relatives of patients with schizophrenia. There is also evidence of a JTC tendency in prodromal groups (Broome et al., 2007).

Recently, there has been considerable interest in the non-competitive NMDA receptor antagonist ketamine as a potential model for schizophrenia. Ketamine infusions in healthy participants have been shown to result in behavioural and cognitive disturbances that are consistent with schizophrenic symptoms (Adler et al., 1999; Krystal et al., 1994; Malhotra et al., 1996). Furthermore, ketamine reliably exacerbates symptoms in patients with schizophrenia, with patients tending to report a re-activation of individual psychotic symptoms under ketamine (Lahti et al., 1995, 2001; Malhotra et al., 1997). Furthermore, glutamate receptor agonists demonstrate therapeutic efficacy (Patil et al., 2007), although evidence of glutamatergic receptor dysfunction in schizophrenia as shown by post-mortem studies is equivocal (McKenna, 2010). Healthy controls receiving ketamine tend not to report the auditory hallucinations often present in schizophrenia, but ketamine reliably induces many negative symptoms including emotional flattening, loss of verbal fluency and impaired memory (Adler et al., 1998; Lahti et al., 2001; Newcomer and Krystal, 2001). Not all negative symptoms are replicated, since ketamine rarely reproduces the disorganized thinking and speech characteristic of schizophrenia, at least not at the doses normally employed by research studies (Lahti et al., 2001; Newcomer and Krystal, 2001). Sensitivity to ketamine varies considerably between individuals. Interestingly, baseline brain activity has been shown to predict vulnerability to ketamine-induced psychosis, and these predictive relations are consistent with symptom-related pathophysiology in schizophrenia (Honey et al., 2008). However, delusional thinking has been reliably reported under ketamine (Corlett et al., 2006; Pomarol-Clotet et al., 2006). A recent study examining recreational ketamine use found increased delusional symptoms in frequent, infrequent, and ex-users, with symptomatology correlating positively with the level of ketamine abuse (Morgan et al., 2009).

Certain prominent theories of delusion formation implicate disrupted processing of reward or error signals (Gray and Snowden, 2005; Hemsley, 2005; Kapur, 2003, 2004; Kapur et al., 2005) and since ketamine has been shown to impact both glutamatergic and dopaminergic systems (Kapur and Seeman, 2002; Moghaddam et al., 1997) this provides a plausible mechanism for delusion formation under ketamine. Specifically, ketamine infusion can lead to enhanced dopamine release in the striatum (Breier et al., 1998; Vollenweider et al., 2000) and dopamine signalling is assumed to represent prediction error and therefore drive associative learning (Schultz, 2002). Furthermore, prediction error responses in lateral prefrontal cortex (PFC) are disrupted by low-dose ketamine such that, under ketamine, PFC activity no longer distinguishes between predicted and unpredicted occurrences (Corlett et al., 2006). Prefrontal responses to mesolimbic input are influenced by glutamate (Lavin et al., 2005), and antagonism of NMDA receptors by ketamine could result in disinhibition of glutamatergic neurons (Moghaddam et al., 1997). Thus, misattributions of reward value under ketamine could lead to certain stimuli becoming highly salient, leading to an early decision and the premature acceptance of a hypothesis, which could result in delusions. Ketamine effects on neural reward circuitry could therefore explain the prevalence of delusional thinking under ketamine.

In the present study, our aim was to investigate how acute ketamine affects performance on the urn task in healthy controls. Since patients have been shown to make early decisions on the urn task, and acute ketamine can induce symptomatology consistent with schizophrenia, we hypothesized that ketamine would lead to a JTC response style in healthy controls. Each participant received two different doses of ketamine plus a placebo condition, and performance on the urn task was tested under each condition. Furthermore, to assess performance under ketamine in relation to its proposed role as a model for schizophrenia, a group of patients was also tested on an identical urn task. A control group not undergoing ketamine infusion (age and IQ-matched to the patients with schizophrenia) was also recruited. By comparing performance between patients and their controls and healthy participants receiving ketamine or placebo, we tested whether ketamine could induce the JTC performance pattern typically observed in patients.

## Methods

### Ketamine group

#### Participants and design

Participants were recruited through an advertisement and were paid for their participation. The study was performed in accordance with the declaration of Helsinki and was approved by the institutional ethics committee (the UCL/UCLH Committee on the Ethics of Human Research). All participants gave written, witnessed, informed consent. The inclusion criteria were that participants were between 18 and 35 years old, with no general health problems and on no regular medications. In all, 20 healthy volunteers (14 male) responded to the advertisement and all took part in this study. Of these, two participants dropped out due to adverse effects: one had a vasovagal episode immediately after intravenous cannulation and one participant vomited after ketamine administration and thus could not continue with testing. Two further participants withdrew midway through the study and their data were excluded from analysis.

**Figure 1.**
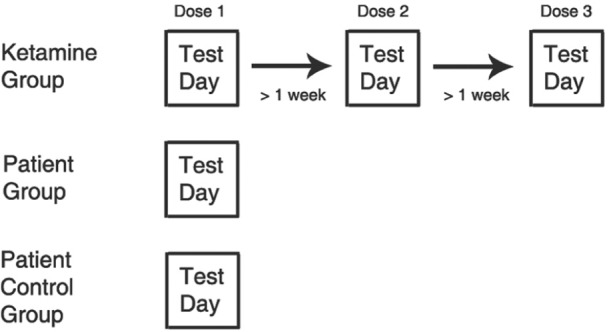
Flow chart depicting test procedure.

Each participant was administered three different ketamine target controlled infusions (see flow chart, Figure 1). These doses were administered on three separate occasions, a minimum of 1 week apart. Participants received the doses in a randomized order; randomization was carried out by the clinical team. Double-blinded procedures were used throughout; only the clinician knew the target dose administered. The procedure was identical regardless of whether ketamine or placebo was delivered, and the researcher administering the task (SE) did not know the dose. Thus all participants underwent testing at each dose of ketamine. Groups were not balanced for gender since more men were recruited. In total, 16 participants completed the study (mean age 25.5 years, SD = 3 years).

#### Drug administration

**Figure 2.**
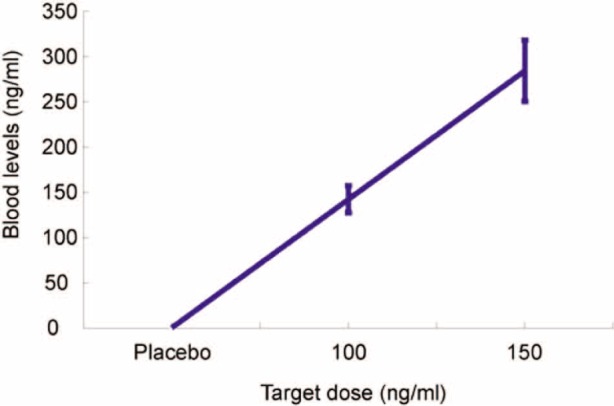
Mean ketamine levels in blood plasma according to target dose.

A 22 gauge intravenous cannula was inserted in the dorsum of the participant’s hand and a ketamine infusion was commenced via a Graseby 3400 intravenous infusion pump controlled by the Stanpump program (Shafer et al., 1990). The program uses a ‘BET’ (bolus–elimination–transfer) infusion scheme that aims to achieve the target plasma concentration almost instantaneously by taking into account ketamine pharmacokinetics using a three-compartment model (Domino et al., 1984). The clinical team employed a similar protocol in a previous research study (Stefanovic et al., 2009). Participants received ketamine aiming for effect-site plasma concentrations of 150 ng/mL, 100 ng/mL and placebo (0.9% NaCl). After behavioural testing was complete a venous blood sample was taken from the antecubital fossa of the other arm to the ketamine infusion. Plasma was obtained immediately from blood samples by centrifugation and samples were stored at -80°C. Ketamine levels were assessed by C3P Analysis (Plymouth, UK). This showed that ketamine levels tended to be in excess of target (Figure 2). *t*-tests confirmed that ketamine plasma levels differed significantly from one another (all pair-wise *t*-tests, *p* < 0.001).

#### Procedure

Testing occurred between 14:00 and 18:00 and the time of testing was broadly matched across groups. Participants arrived at the hospital after completing a 6-h fast.

The anaesthetist commenced the infusion after intravenous cannulation. Throughout the infusion, the participant’s pulse, blood pressure, oxygen saturation and electrocardiogram (ECG) were monitored. At the end of each session, participants were assessed by medical staff as to their ‘street readiness’. Participants were given a contact telephone number for the clinical team in case of adverse effects after departure; none were reported.

### Patient group

We recruited 39 individuals who met the DSM-IV criteria for schizophrenia from the outpatient department of South London and Maudsley NHS Trust. Patients were stable on treatment with antipsychotic medication (Table 1), those with dual diagnoses and drug and alcohol problems were excluded from the study. Patients underwent a Positive and Negative Syndrome Scale (PANSS) diagnostic interview on the day of testing; demographic details and PANSS scores are given in Table 1.

**Table 1.** Participant demographic information.

	Patient group (*n* = 39, Male = 30)		Control group (*n* = 39, Male = 26)		Ketamine group (*n* = 16, Male = 12)	
	Mean (s.d.)	Range	Mean (s.d.)	Range	Mean (s.d.)	Range
Age	38.84 (9.35)	21–61	34.24 (13.23)	18–61	25.5 (3)	24–34
IQ	104.9 (11.8)	81–128	112.2 (13.0)	75–127		
PANSS score						
Positive	13.89 (6.03)	7–26				
Negative	13.81 (6.09)	7–34				
Total	52.45 (15.58)	30–89				
Medication						
Chlorpromazine Equivalents (mg/day)	325 (225)*	100–1000				

* These data were unavailable for five of the participants.

### Patient control group

Some 39 control participants were also recruited, and these were age and IQ matched to the patient group. Inclusion criteria stipulated no psychiatric history and no history of drug/alcohol problems. For demographic information see Table 1.

### Task

All groups received the same test materials, which were delivered via computer. For the ketamine group, testing commenced once the clinician was satisfied that the dose levels had stabilized. All groups received the same instructions, on-screen, prior to testing. The task followed the standard procedure for the ‘urn’ task (Huq et al., 1988). Participants were told that they would be shown beads from two ‘urns’ containing blue and red beads in a 85/15 and 15/85 ratio. After each bead was drawn and shown to the participant, the participant indicated whether they wanted to draw again, or guess the urn. During the task, for each sequence, a series of beads (represented by coloured discs on-screen) was presented to the participant. The participant used the keyboard after every presentation to indicate whether (a) s/he would like to be shown another bead or (b) make a decision as to the ‘urn’, and if so, which ‘urn’ to choose. Only the last bead was shown on the screen. The series of presentation was pre-determined. Five different sequences were presented. Sequence order was randomized both within (i.e. across sessions) and between subjects. The sequences were:

Sequence 1: [R R R R R R R R R R]

Sequence 2: [R B R R R R R R R R]

Sequence 3: [B B R B B B B B B B]

Sequence 4: [R B B B B B B B B B]

Sequence 5: [B B R R R R R R R R]

In the ketamine group, participants completed the task on each of the three test days. In the patient, and patient control groups, participants were tested once (see flow chart, Figure 1). In the ketamine group, participants were cannulated on all of the test days. Patients and patient controls were not cannulated on their test day.

### Data analysis

In line with previous work employing this task, the dependent variable was ‘draws to decision’; we calculated the average number of beads shown per sequence before a decision was made. This applied to all groups of participants. For the ketamine group, a one-way analysis of variance (ANOVA) with random effects was employed to test the hypothesis that dose level as a within-subject factor had a significant effect on mean draws to decision. A one-way ANOVA was also used to test for a difference between patients and matched control groups, except in this case the group effect was between subject. Data from patients, matched controls, placebo and high dose ketamine were then entered into a single one-way ANOVA. In this case, the ketamine dose was not treated as a repeated measure but rather as a between-subject factor. A contrast was specified over these four groups to compare differences between patients and controls with differences between placebo and high dose ketamine. SPSS was used for all analyses.

## Results

The initial analysis examined within-subjects data in the ketamine group, and a between-subjects comparison between the patients and matched controls.

**Figure 3.**
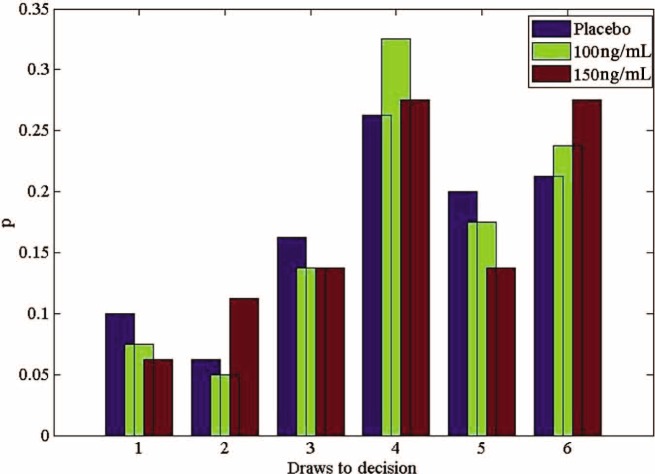
Probability distributions of ‘draws to decision’ under ketamine. Pooled data across all participants, according to dose.

**Figure 4.**
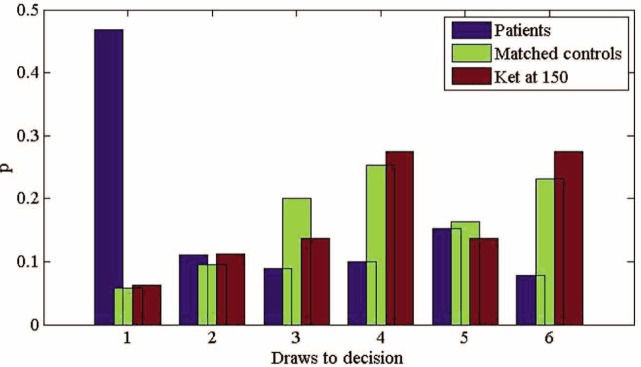
Probability distributions of ‘draws to decision’ for patients, matched controls, and controls receiving ketamine at the higher dose.

For the ketamine group, an ANOVA on the mean number of draws showed no effect of ketamine dose (F(2,45) = 0.08, *p* = 0.92). Thus, participants made approximately the same number of draws under each dose of ketamine (Figure 3). When the same analysis was carried out on the patients and their matched controls, we found a significant main effect of group (F (1,76) = 18.64, *p* < 0.001). Similar to previous studies, patients required fewer draws before they inferred the urn than controls (Figure 4).

Subsequent to this, an additional ANOVA was used to compare data from four groups. Data from patients, matched controls, ketamine group (placebo) and ketamine group (high dose) were entered into a single one-way ANOVA. It was found that performance differences between patients and their matched controls were larger than the difference between participants receiving ketamine (150 ng/mL) and placebo. A contrast was specified which tested the value of (patients - controls) - (high dose - placebo). This contrast was found to be significant (*t* (106) = 2.187, *p* = 0.031). Thus, the differences between each pair of groups (as specified in the contrast) were themselves different from each other.

## Discussion

Previous studies have shown that ketamine can model some aspects of schizophrenic symptomatology (Adler et al., 1999; Krystal et al., 1994; Malhotra et al., 1996). Other work has shown, reliably, that patients with schizophrenia show a JTC reasoning bias in probabilistic inference tasks (Averbeck et al., 2011; Garety and Freeman, 1999; Huq et al., 1988). Deluded patients show this bias even when a memory aid is included in the ‘urn’ task, suggesting that memory difficulties are not responsible (Dudley et al., 1997). While we were able to replicate the JTC bias in patients relative to matched controls, we found no evidence that ketamine made healthy controls adopt a JTC reasoning bias. It could be argued that the dosages employed here were insufficient since some previous studies (which reported high levels of delusional ideation) used a higher dose of 200 ng/mL (Corlett et al., 2006; Pomarol-Clotet et al., 2006). However, we found that our actual blood levels were significantly higher than the target, in some cases exceeding 200 ng/mL. Therefore, we do not believe that insufficient ketamine levels can account for lack of an effect. We adopted a lower target dose because pilot testing at higher doses led us to believe that the level of drop-out would be high; furthermore, at 200 ng/mL target dose, our pilot participant was physically unable to perform the task.

Ketamine has been shown to promote delusions and other ‘positive’ symptoms in healthy controls (Corlett et al., 2006; Pomarol-Clotet et al., 2006), and worsen the psychotic symptoms of patients (Lahti et al., 1995, 2001; Malhotra et al., 1997). Some theories of delusion formation have related JTC to disruptions in either reward (Kapur, 2003, 2004; Kapur et al., 2005) or prediction error (Gray, 1995) signals, and ketamine has been shown to influence these signals (Corlett et al., 2006; Vollenweider et al., 2000). Nevertheless, we have found that performance on the urn task is not affected by ketamine, suggesting that perhaps JTC does not result directly from abnormal dopaminergic or glutamatergic signalling. This ties in with behavioural studies comparing responses in the urn task with other probabilistic inference tasks, showing that patients who jump to conclusions actually learn less from rewarding stimuli than patients who gather more information (Averbeck et al., 2011). This does not support an increased salience or increased reward prediction error hypothesis of JTC, instead suggesting a decreased threshold for making inferences. This does not imply simple impulsivity, however, since patients adjust their behaviour if task difficulty is increased, requiring more draws to decision (Dudley et al., 1997). It is possible that increased tonic dopamine levels in patients with schizophrenia are driving them to respond quickly, and that ketamine does not increase tonic dopamine levels. This, however, remains an open question.

The results of the current study suggest that ketamine infusion does not replicate in controls the JTC tendency observed in patients. Ketamine certainly does not induce all aspects of schizophrenia symptomatology, since certain negative symptoms in particular are largely absent; nevertheless, ketamine tends to provoke a wider range of psychotic symptoms compared with amphetamine (Lahti et al., 2001). However, although ketamine delivery does exacerbate psychotic symptoms in patients, there are qualitative differences in ketamine effects between patients and controls (Malhotra et al., 1997). The data reported here suggest that the JTC aspect of schizophrenic cognition is not reproduced by ketamine, and raises the possibility that delusion formation under ketamine is not underpinned by JTC. Nevertheless, certain important caveats should be noted. The urn task employed here explores one specific case of probabilistic inference. Therefore the results reported here do not imply that ketamine has no effect on probabilistic inferences, since a more comprehensive investigation is required. Disruptions to fronto-striatal activity caused by ketamine might not be sufficient to alter behaviour in the urn task. A more sensitive measure may be required. Although a JTC style of reasoning has been demonstrated in patients using other tasks (Kemp et al., 1997; Linney et al., 1998), it seems that the task structure might be critical. Specifically, it has been suggested that stress induced by perceived time pressure in the urn task might cause patients with schizophrenia to appraise stimuli inaccurately (Glöckner and Moritz, 2009), although this needs to be more fully explored. Also, studies of executive function under ketamine point to task-specific impairments (Honey et al., 2003; Krystal et al., 2000), raising the possibility that participants altered their cognitive strategy to perform as normal on the urn task under ketamine. Further work employing more challenging probabilistic inference tasks would be useful for exploring these possibilities. One could, for example, increase the difficulty of the urn task (since JTC might manifest only under certain levels of task difficulty) or use a more in-depth assessment tool such as Mouselab (Payne et al., 1988) to investigate decision strategies. In addition, a recent study has shown that certain cognitive changes relating to delusion proneness are only detectable following chronic administration of ketamine (Freeman et al., 2009). Although recruitment of recreational drug users presents certain difficulties (since most are polydrug users, for example), this might provide a worthwhile insight into the cognitive changes induced by ketamine use.

## Conclusion

We have found that acute administration of the NMDA antagonist ketamine does not appear to replicate in controls the JTC response pattern frequently observed in patients with schizophrenia. Further work using other probabilistic inference tasks would be beneficial, as would investigations in chronic ketamine users. The characterization of reasoning biases in patients receiving ketamine would also be informative.
